# Jungfraujoch: hardware-accelerated data-acquisition system for kilohertz pixel-array X-ray detectors

**DOI:** 10.1107/S1600577522010268

**Published:** 2023-01-01

**Authors:** Filip Leonarski, Martin Brückner, Carlos Lopez-Cuenca, Aldo Mozzanica, Hans-Christian Stadler, Zdeněk Matěj, Alexandre Castellane, Bruno Mesnet, Justyna Aleksandra Wojdyla, Bernd Schmitt, Meitian Wang

**Affiliations:** a Photon Science Division, Paul Scherrer Institute, Forschungsstrasse 111, 5232 Villigen, Switzerland; b Scientific Computing, Theory and Data Division, Paul Scherrer Institute, Forschungsstrasse 111, 5232 Villigen, Switzerland; c MAX IV Laboratory, Lund University, Fotongatan 2, 221 00 Lund, Sweden; d IBM France, 21 av Simone Veil, 06206 Nice, France; RIKEN SPring-8 Center, Japan

**Keywords:** X-ray detectors, macromolecular crystallography, X-ray image acquisition, data acquisition, field-programmable gate arrays (FPGAs)

## Abstract

The JUNGFRAU 4-megapixel (4M) charge-integrating pixel-array detector, when operated at a full 2 kHz frame rate, streams data at a rate of 17 GB s^−1^. To operate this detector for macromolecular crystallography beamlines, a data-acquisition system called Jungfraujoch was developed. The system, running on a single server with field-programmable gate arrays and general-purpose graphics processing units, is capable of handling data produced by the JUNGFRAU 4M detector, including conversion of raw pixel readout to photon counts, compression and on-the-fly spot finding. It was also demonstrated that 30 GB s^−1^ can be handled in performance tests, indicating that the operation of even larger and faster detectors will be achievable in the future. The source code is available from a public repository.

## Abstract 2

A new data acquisition and real-time image analysis system with FPGAs and GPUs for kilohertz macromolecular crystallography applications is presented.

## 1. Introduction

The development of X-ray detectors has assisted groundbreaking science at synchrotron macromolecular crystallography beamlines. Readout noise, dynamic range, energy coverage and frame rate have been continuously improved with each new generation of detectors. This was experienced at the Paul Scherrer Institute (PSI) with PILATUS (12.5 Hz) and EIGER 16M (133 Hz), introduced in 2007 and 2015, respectively (Broennimann *et al.*, 2006 ▸; Dinapoli *et al.*, 2011 ▸), which resulted in increasing diffraction resolution with fine slicing (Mueller *et al.*, 2012 ▸; Casanas *et al.*, 2016 ▸; Förster *et al.*, 2019 ▸), enabling experimental phasing with native elements (Basu *et al.*, 2019 ▸) and fast data collection for fragment-based screening (Thomas *et al.*, 2019 ▸; Kaminski *et al.*, 2022 ▸) and serial crystallography (Diederichs & Wang, 2017 ▸). The upgrades of third-generation synchrotron radiation facilities toward diffraction-limited storage rings like the upcoming Swiss Light Source (SLS) 2.0 will increase source brilliance further, calling for continuous development of detectors and readout systems operated at kilohertz and higher frame rates. For example, the JUNGFRAU integrating detector (Mozzanica *et al.*, 2018 ▸), which is not count-rate limited and can operate at 2 kHz frame rate (Leonarski *et al.*, 2018 ▸), has been recently commissioned at the SLS.

**Figure 1:**
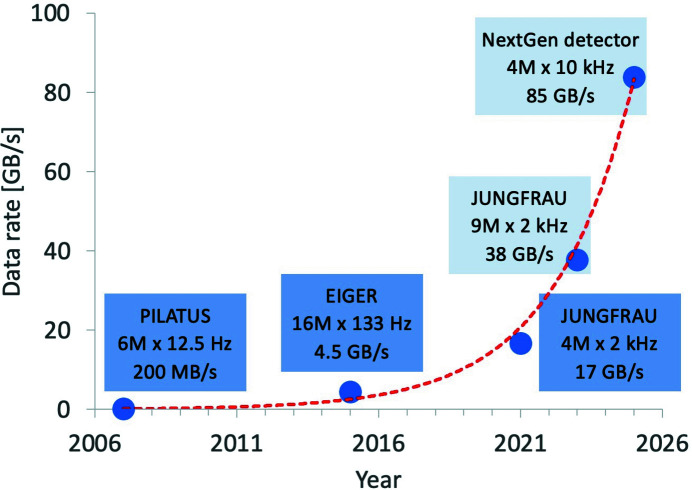
Data rates at the SLS MX beamlines from the introduction of PSI PILATUS in 2006 to post-SLS 2.0 upgrade prediction – the trend is showing an exponential increase in data rates over the years. The *x* axis displays the year of introduction of the detector to the beamline, while the *y* axis shows raw data rates.

Since the advent of the PILATUS detector in 2007, data rates have increased exponentially over time, doubling every two years. This trend will continue (see Fig. 1 ▸) with next-generation detectors and next-generation synchrotrons (Denes & Schmitt, 2014 ▸). With a limited natural increase in computing hardware performance (Hennessy & Patterson, 2019 ▸), it is unrealistic to expect that existing software-based acquisition applications will catch up with faster detector data rates. There exist two possible solutions to tackle this issue: either massive parallelization or a change of paradigm from mainstream central processing units (CPUs) to task-specific architectures, such as general-purpose graphics processing units (GPGPUs) or field-programmable gate arrays (FPGAs). The former approach may seem easier to realize, but increasing the number of servers to match exponential growth is unsustainable in the long term. It can be safely expected that task-specific architectures will advance rapidly in the future, mainly thanks to their higher power efficiency and their growing popularity within the machine-learning community. We therefore believe that embracing such task-specific architectures is an attractive, sustainable and forward-looking approach.

In this article, we provide an example that adopting advanced tools, like coherent OpenCAPI (Stuecheli *et al.*, 2018 ▸; Hoozemans *et al.*, 2021 ▸) interconnect and high-level synthesis, simplifies FPGA development within the synchrotron context. We also present Jungfraujoch, a detector readout system that can handle a continuous data stream of 17 GB s^−1^ from a 2 kHz JUNGFRAU 4-megapixel (4M) detector within a single two-rack-unit server box, facilitating high data-rate scientific applications.

## 2. Jungfraujoch architecture

The key element of Jungfraujoch is an IBM IC922 server equipped with FPGAs and inference-grade GPGPUs, which receives, sorts, reduces and buffers data from the JUNGFRAU detector. The task is achieved with a combination of FPGA design and software algorithms, with a minor part accelerated on a GPGPU. The IBM architecture was chosen for its superior memory bandwidth and the coherent OpenCAPI interconnect, which significantly reduces the complexity of integrating FPGA design with the software.

**Figure 2:**
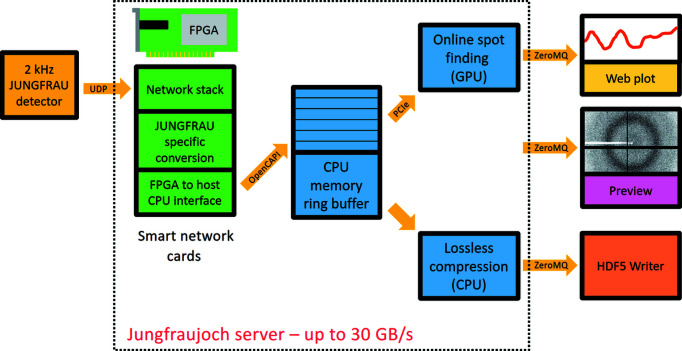
The Jungfraujoch data flow from the JUNGFRAU detector, via the FPGA card to the CPU buffer for compression and spot finding.

While memory-bandwidth-demanding tasks of Jungfraujoch are handled on the IBM server (see Fig. 2 ▸), the system is designed with auxiliary services, like writer and broker, which can run either on the IBM server or on another machine connected via a fast network. For example, to ensure the compatibility of servers constituting a high-performance file-system cluster, file writing can be performed on an external node from the facility’s preferred vendor. The control of a distributed architecture, spanning (potentially) multiple machines, is realized with *Google Remote Procedure Call* (*gRPC*) along with *Google Protocol Buffer* (*Protobuf*) serialization layer. The *gRPC* and *ProtoBuf* enable seamless integration of code written in multiple programming languages. The core of Jungfraujoch is written in C++ and interacts with beamline control applications, which are usually written in Python and JavaScript web front-end applications. Technical details are given in Appendix *A* [App appa].

### 2.1. FPGA smart network card

The critical part of data acquisition is the first step, where a large amount of data is received from the detector. Any delay in processing can result in lost packets, as the detector is not able to re-transmit missed data (fire and forget mode). Real-time performance is guaranteed with the FPGA’s deterministic throughput. Another advantage of FPGAs is embedded input/output capability, primarily via 100 Gbit s^−1^ ethernet, so that the network traffic does not have to travel via CPU memory to be ingested into the FPGA fabric.

The FPGA design for Jungfraujoch consists of three major parts (see Fig. 2 ▸), which are described below.

#### 2.1.1. Network stack

The physical network layer is handled by a 100 Gbit s^−1^ media-access-control hard (built-in) core embedded in a Virtex Ultrascale+ FPGA. Next, the data stream passes through an ethernet data link, internet protocol, user datagram protocol (UDP) and JUNGFRAU PSI detector header handlers, which are based on an open-source design (Sutter *et al.*, 2018 ▸; Ruiz *et al.*, 2019 ▸). Only the receiving part of the protocol stack is implemented. The network stack ends with a finite-state machine, which makes it possible to switch between the idle state, when packets are discarded, and the receiving state, when packets are forwarded through the pipeline.

As testing the FPGA pipeline with an X-ray detector at a source of high-intensity X-rays is not always convenient, Jungfraujoch has built-in functionality to internally create a flow of packets. The core has 1 MB of memory that can be preloaded from host memory, at the beginning of the operation, with data for a single detector module (512 × 1024 pixels).

#### 2.1.2. Conversion from raw readout to photon counts

As the JUNGFRAU is an adaptive gain detector it is necessary to transform raw readout values into photon counts, or accumulated energy, before data analysis. This procedure has been described in prior publications (Redford *et al.*, 2018*a* ▸; Leonarski *et al.*, 2020 ▸). It includes subtraction of dark current (pedestal), multiplication by gain factor and multiplication by 1/X-ray photon energy.

All calculations are expressed in fixed-point arithmetic, which reduces both FPGA logic utilization and bit width of operands. All coefficients and photon-count results are 16-bit integers (Leonarski *et al.*, 2020 ▸). Within the standard energy range of 4–20 keV for macromolecular crystallography application, this procedure gives differences of less than 0.30 photons between fixed point and ideal floating point, while 0.25 photons are expected for truncation of the floating-point result to 16-bit integer. The impact of this procedure on data quality for macromolecular crystallography is expected to be negligible, as discussed previously (Leonarski *et al.*, 2020 ▸).

For each pixel, *i.e.* for 16 bits of raw data, six correction constants (three gain factors and three pedestals) have to be loaded from memory, requiring memory bandwidth to be at least six times the incoming stream. Handling such challenging throughput requires high bandwidth memory (HBM) equipped FPGAs (Wang *et al.*, 2020 ▸), available with up to 460 GB s^−1^ memory bandwidth at the date of this publication.

To help in downstream compression, we implemented an optional pre-compression *bitshuffle* filter (Masui *et al.*, 2015 ▸) on the FPGA. It has a fixed block size of 4096 pixels × 16 bits, corresponding to a single detector packet. Applying the filter on the FPGA precludes further transformations and analysis on CPUs unless the filter effect is reverted in software. Thus the FPGA *bitshuffle* filter is generally disabled by the software pipeline.

#### 2.1.3. FPGA to host CPU communication

The main communication channel is a large host (CPU) memory buffer, where images are written by the FPGA card and are read by a software service for further analysis. The buffer is divided into locations, each accommodating one detector module (512 × 1024 pixels × 16 bits).

The way to communicate the status of the buffer between the FPGA and software is similar to remote-direct-memory-access adapters with work request (WR) and work completion (WC) queues. In this mechanism, the software informs the FPGA about free buffer locations by posting a WR with a location number and a 64-bit memory address. When the FPGA encounters a new frame number for a module, it reads the WR queue and starts transferring packets to the given memory location. When frame measurement is finished (the actual condition is arrival of the *N* + 2 frame or end of the acquisition; this is to allow for some degree of packet reordering), the FPGA posts a WC to the queue. This WC includes location number, indication if all packets were received, and metadata, *e.g.* frame number, module number and timestamp from the detector header. The software polls the WC queue to know when to start processing the images.

This design guarantees exclusive access to the buffer for either FPGA cards or software. It also allows for flow control in case the software side of Jungfraujoch is not able to cope with incoming data from detector modules – the WR queue becomes empty and FPGA design pauses (stalls) until the software is ready to receive new packets. Incoming packets are then buffered within the FPGA, roughly up to 4 MB in size. If this capacity is exceeded, further packets are dropped, resulting in missed frames, until congestion is resolved.

The advantage of our solution is a significant reduction of control overheads. For Jungfraujoch, the CPU and FPGA exchange control information per one complete detector module, *i.e.* 1 MB. On the other hand, a standard network card has to signal the CPU for every received ethernet packet. In the JUNGFRAU detector case, this one packet size is 8 kB, so it is 128 times higher frequency of control messages.

#### 2.1.4. FPGA design tools

FPGA development has benefited from two important technologies: high-level synthesis and OpenCAPI.

The smart-network-card FPGA design is written primarily in C++, which is different from standard practice in hardware design. Naturally, digital circuits are designed with hardware description languages (HDLs), like Verilog or VHDL, which make it possible to describe the inherent parallelism of hardware. While HDLs allow one to obtain the most optimal design, they are time consuming in development and require a set of skills uncommon for software developers. Therefore, FPGA vendors provide high-level synthesis tools that transcompile C++ code, with some additional directives (pragmas), into HDL. One of the advantages of high-level synthesis is to guarantee that the behavior of C++ code and resulting HDL is close enough – therefore initial testing can be performed directly on C++ code.

**Figure 3:**
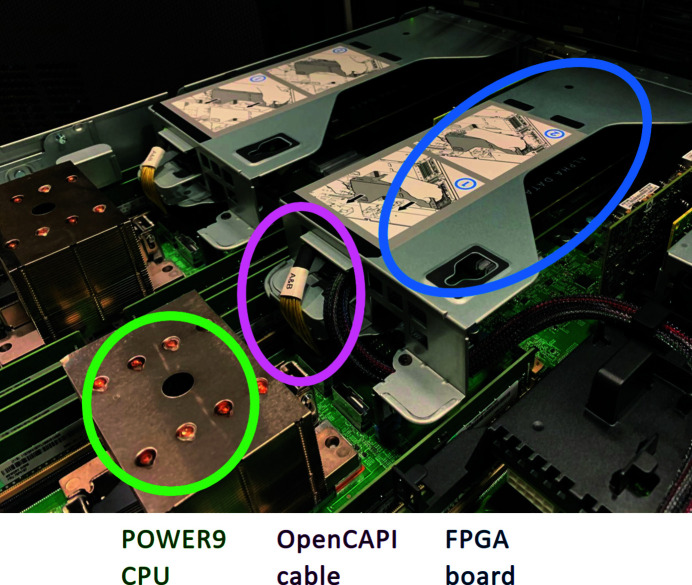
A photograph of the IC922 server (IBM). The OpenCAPI interface over a dedicated cable allows the FPGA to coherently access the memory of the host CPU.

OpenCAPI (Stuecheli *et al.*, 2018 ▸; Hoozemans *et al.*, 2021 ▸) is a new-generation interconnect (see Fig. 3 ▸), currently only available in IBM Power architecture servers. The OpenCAPI features full memory coherence, *i.e.* the connected device sees memory the same way as a program running on the host CPU, making it possible to benefit from virtual addressing present in modern CPUs. The OpenCAPI relieves developers from taking care of the low-level details of memory hierarchy on the operating system level and allows them to focus on high-level productivity. A key feature of OpenCAPI is the possibility of running design simulations with both FPGA design and the actual software component via the *OpenCAPI Simulation Engine*. The OpenCAPI nominal throughput is 25 GB s^−1^, corresponding to 2 × 100 Gbit s^−1^ ethernet links. This is more throughput than PCI Express, currently the most commonly used peripheral bus, which has a nominal bandwidth of 16 GB s^−1^ (Gen3x16 present in Xilinx Ultrascale+) and has a lower actual performance due to communication overheads (Nakamura *et al.*, 2017 ▸; Durante *et al.*, 2015 ▸).

### 2.2. Jungfraujoch software service

The FPGA card is integrated with the beamline software stack via a Jungfraujoch software service. The software service is a multi-threaded application that handles data from the detector via FPGAs and composes full images from detector modules with optional summation. Images are then compressed and forwarded via a ZeroMQ PUSH socket to the HDF5 writer. To improve performance, it is also possible to send images over multiple PUSH sockets on a round-robin basis.

The Jungfraujoch software service provides a subset of compression options to the user. *Bitshuffle* filter (Masui *et al.*, 2015 ▸) is always applied, however in the later stage it can be combined with the default *LZ4* or an alternative *Zstandard* (Facebook) compressor. The latter can provide a better compression ratio for X-ray diffraction images at the cost of lower performance (Leonarski *et al.*, 2020 ▸). As a third choice, a custom experimental compressor was developed for Jungfraujoch, which limits itself to a run-length encoding of two characters, 0 and 255 (all 0 bits or all 1 bits), producing *Zstandard* compliant format (Collet & Kucherawy, 2021 ▸) output. This is based on properties of X-ray diffraction images, which after *bitshuffle* filtering show long sequences of zeros.

To provide quick feedback, the Jungfraujoch software service also performs the following three additional operations on a subset of images:

(*a*) Uncompressed images are published at a default rate of one image per second via a ZeroMQ PUB socket for display on a beamline console providing visual live feedback.

(*b*) Basic image statistics, like mean count and radial integration profile within a resolution range, are calculated at a default rate of 100 images per second. Results are saved and can be enquired via *gRPC*, for example to show plots on a web page. This feature can be useful as diagnostics and fast experimental feedback, *e.g.* to analyze and visualize changes in scattering background of the diffraction image.

(*c*) Spot-finding analysis is performed to estimate the number of diffraction peaks in the image, thus enabling assessment of the overall quality of diffraction – the default is to run this analysis at a rate of 100 images per second. We implemented the COLSPOT algorithm (Kabsch, 2010 ▸) for its relative simplicity and robustness. The part of the algorithm that is most compute-intensive, *i.e.* selection of strong pixels, was accelerated on a GPGPU. This way, spot finding is not competing for CPU cycles with compression, reducing the likelihood of lost frames. Results of the spot finding, *i.e.* the list of reciprocal-space points corresponding to spots, are published via a ZeroMQ PUB socket for an external indexing routine.

The Jungfraujoch software service also calculates dark current (pedestal) values and root mean square deviation of the pedestal, according to a previously described procedure (Redford *et al.*, 2018*a* ▸; Leonarski *et al.*, 2020 ▸). Pedestal calculation is accelerated with a GPGPU.

### 2.3. Example integration applications

Jungfraujoch is designed to be part of a beamline software infrastructure; therefore it has to be easily integrable into larger frameworks, such as *LIMA* (Petitdemange *et al.*, 2018 ▸) for image processing, *MXCube* (Oscarsson *et al.*, 2019 ▸), *GDA* (Gibbons *et al.*, 2011 ▸), *DA+* (Wojdyla *et al.*, 2018 ▸), *BLISS* (Michel *et al.*, 2019 ▸), *Karabo* (Hauf *et al.*, 2019 ▸) and *Bluesky* (Allan *et al.*, 2019 ▸) for control and synchronization. Below we provide a list of example applications that allow its standalone operation. Because of the modular nature of the design, these applications can be easily replaced with alternatives in accordance with synchrotron facility preferences.

(i) ‘*gRPC* broker’ is a state machine that controls the behavior of the whole environment. It presents the user with options to start, stop and cancel measurements, as well as to display the current status of services.

(ii) ‘HDF5 writer’ receives image chunks via ZeroMQ and writes them into a data file. Given that the Jungfraujoch service already compresses the chunks, the HDF5 writer requires very little resources. The system allows multiple writers to operate in parallel, assuming that they manipulate different files. The HDF5 writer is also responsible for writing a metadata ‘master’ file according to the NXmx Gold Standard (Bernstein *et al.*, 2020 ▸).

(iii) ‘Indexer application’ is a wrapper over *XGANDALF* (Gevorkov *et al.*, 2019 ▸) or other indexing algorithms, which processes diffraction spot positions received via ZeroMQ.

(iv) ‘Detector application’ is a wrapper over the *SLS Detector Package*, allowing configuration of the JUNGFRAU detector.

(v) ‘Preview application’ is a Python script that receives images by subscribing to a ZeroMQ publisher and displays them in the DECTRIS *Albula* diffraction viewer.

## 3. Performance results

### 3.1. Nominal Jungfraujoch FPGA performance

One of the advantages of the FPGA is that performance can be inferred from the design itself. The FPGA is designed to work with a 250 MHz clock. Single transfer size in the pipeline is 64 byte (512 bit), determined by an output of 100 Gbit s^−1^ ethernet core. Each component is devised to accept one packet per clock cycle (initiation interval), therefore nominal throughput of the in-FPGA processing is 64 byte × 250 MHz = 16 GB s^−1^.

The maximal throughput of data ingest via a single 100 Gbit s^−1^ ethernet port is 12.5 GB s^−1^, and maximal data transfer to the host system via a single OpenCAPI link is 25 GB s^−1^. As total performance is determined by the slowest element of the pipeline, for an FPGA card with only a single 100 Gbit s^−1^ port enabled, the performance is ultimately limited to 12.5 GB s^−1^ for a network-connected system. For testing purposes, a higher rate of 16 GB s^−1^ is possible, where images are internally generated within the FPGA.

### 3.2. Measured Jungfraujoch performance

To verify the performance of the system, we prepared a routine that runs both FPGA and Jungfraujoch software routines, with data provided by the in-FPGA internal packet generator. In this way, all internal steps of the pipeline are tested, specifically the throughput of the FPGA itself, the throughput of the OpenCAPI interconnect, geometry transformation, compression and spot finding. As a sanity check, the last image is compared with the expected values. Naturally such a test excludes external communication, *i.e.* the FPGA receiving UDP packets from the detector and the efficiency of the HDF5 writer.

The test was executed on an IBM IC922 server equipped with two 20-core POWER9 CPUs, two Alpha Data 9H3 FPGA boards and two Nvidia T4 GPUs. Eighty compression threads were conducted in parallel, while spot finding and radial integration analyzed every 20th frame. Data-collection size was set to one million frames, ensuring that local buffering does not affect results. To ensure realistic compression factors, we loaded a typical lysozyme diffraction image to FPGA memory and simulated each card receiving data from four detector modules with the internal packet generator. Assuming that the pipeline (both FPGA and software) is paused when downstream processing is unable to match the performance speed, the frame rate will equilibrate at the rate of the slowest component.

Results of the measurement are presented in Table 1 ▸ and show that the system has a significant margin over the target of 4M at 2 kHz performance, reaching above a 3 kHz frame rate. This demonstrates that the Jungfraujoch server has the potential to support even larger detector systems in the future. Moreover, our results illustrate that handling the highest data rates requires custom compression developments, as the Jungfraujoch *Zstandard* implementation outperformed the standard *LZ4* compressor.

During this test, we monitored environmental sensors within the server. The total server power consumption during the test was ∼680–690 W, compared with 330 W when idle. FPGA board power consumption was in the range of 32–25 W per board, based on voltage and current sensor readout (Alpha Data Board Control Interface; sum for 3.3 V and 12 V rails), while power consumption per GPU was in the range of 30–40 W (*nvidia-smi* utility).

## 4. Conclusions and outlook

The Jungfraujoch system described in this article is a prime example that task-specific computing architectures can be successfully utilized for data acquisition with high-frame-rate pixel-array detectors. Currently, the system can handle 30 GB s^−1^ data from JUNGFRAU detectors at the maximum frame rate of 2 kHz and is a solid basis for the future development, such as a higher-performing framework aimed at 10 kHz detectors.

One of the significant achievements of our design is the compact size of the whole system. Main components, *i.e.* data acquisition, compression and image analysis (*e.g.* spot finding), are all packed into a single two-rack-unit server system. Such design, inspired by the DECTRIS Detector Control Unit, enables easy transport or system copy. This allowed us to test and gain experience from operating the Jungfraujoch system at three synchrotron beamlines: X06SA (SLS/PSI), BL-1A (Photon Factory, Japan) and BioMAX (MAX IV Laboratory, Sweden). The results of these beam times will be published separately.

Maximizing the functionality within a single server was possible thanks to the high power efficiency of task-specific accelerators (FPGAs and GPGPUs). For example, the throughput of a single FPGA board in converting JUNGFRAU images from raw to photon count representations exceeds the performance of a four-socket CPU server used by us in a previous work (Leonarski *et al.*, 2020 ▸). This is a 30-times increase in power efficiency, given the server consumption was above 1 kW and the FPGA board was at 30 W.

Yet, the power efficiency would not be enough if programming the accelerators required prohibitively high effort. FPGA high-level synthesis and OpenCAPI interconnect are two technologies that have made the Jungfraujoch system development feasible. At the initial stages of the project, the benefits of using these features were the shortening of development time and the reduction of the entry barrier for FPGAs software development. With the system’s complexity growing over time, the simplicity and robustness of design verification became advantageous. To increase the accessibility of Jungfraujoch, we are currently working on a PCI Express implementation. Given most parts of the current design can be reused thanks to standard FPGA interfaces, such as the Advanced Extensible Interface (AXI), we expect the two flavors of the design to coexist. OpenCAPI design would allow for rapid development, prototyping and handling of more advanced scenarios, while PCI Express would make deployment possible on a broader range of hardware platforms.

Our results demonstrate the benefit of combining data acquisition with real-time image analysis within a single server system. If the calculations were performed on an external system, this would increase image compression, transfer and decompression overhead. It is especially the case for image-analysis methods with a small performance footprint, like radial integration or spot finding. The current implementation can analyze only a subset of images in real time at the maximum frame rate. While this is good enough for experimental feedback, we aim to scale up analysis performance for every image without affecting the frame rate for the next iteration of the Jungfraujoch.

Task-specific FPGA and GPGPU architectures will develop rapidly in the future, especially in the context of ongoing advances in machine learning, given that these techniques perform exceptionally well on dedicated accelerators. As it is likely that future X-ray diffraction image analysis (Ke *et al.*, 2018 ▸) or compression (Roy *et al.*, 2021 ▸) is going to be based on neural networks, we also expect that the next version of the Jungfraujoch system will be designed to accommodate machine-learning algorithms as part of the pipeline.

## Figures and Tables

**Figure 4:**
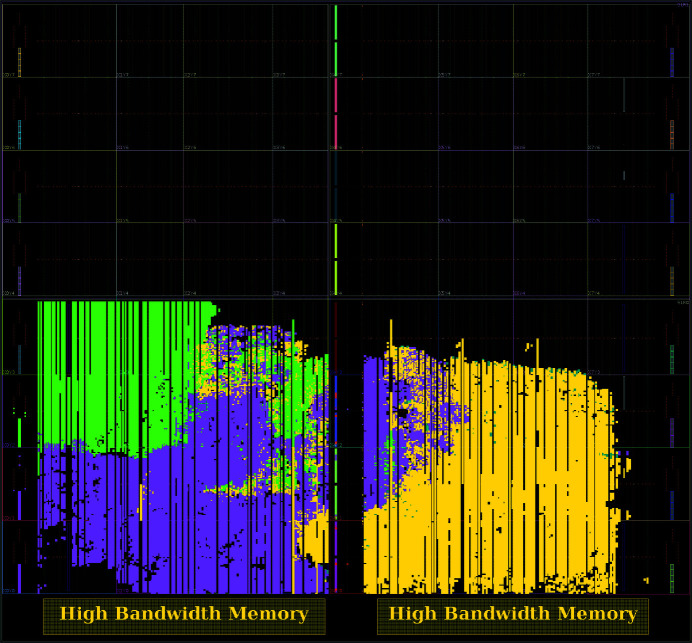
This figure shows how functionalities of Jungfraujoch are placed on the XCVU35P device by the FPGA synthesis tool: network stack (green), host-memory interface including OpenCAPI (purple) and JUNGFRAU specific conversion routines (yellow). The black region, mostly in the top half of the FPGA, is not occupied, leaving space for further functionality. The figure is produced by *Xilinx Vivado* 2022.1 after an implementation run.

**Table 1:** Performance results for the Jungfraujoch FPGA and software show that the system has a significant margin over 2 kHz and potentially can reach a performance above 3 kHz Presented numbers are from test handling one million images generated with the FPGA internal packet generator, corresponding in size to a 4M detector.

Compression algorithm	Execution time (s)	Throughput (GB s^−1^)	Frame rate (kHz)	Compression factor
*Bitshuffle*/*Zstandard* (Jungfraujoch compressor)	273	31	3.6	5.1
*Bitshuffle*/*LZ4*(standard compressor)	320	26	3.1	5.7

**Table 2:** FPGA resource utilization is relatively low for the XCVU35P chip, allowing for more functionality to be implemented in the future Data from the *Xilinx Vivado* 2022.1 tool, after synthesis and implementation.

Resource	Count used	Share of available resources
Configurable logic block	40654	37%
Look-up table	169765	19%
Flip-flop register	294165	17%
Digital signal processor	162	3%
Block RAM	1.2 MB	20%
Ultra RAM	6.2 MB	28%
HBM interface	12	37.5%

## Appendix A Jungfraujoch technical details

### A1. Hardware requirements

Jungfraujoch is designed to operate on an IBM IC922 system. The following configuration was used for tests at PSI: two 20-core POWER9 CPUs (2.9–3.8 GHz), 256 GB RAM, two Alpha Data 9H3 FPGA boards with Xilinx Virtex Ultrascale+ HBM FPGA (first with XCVU33P, later with XCVU35P chip), two Nvidia T4 GPGPUs and a single Mellanox Connect-X 5 100 Gbit s^−1^ ethernet adapter. Amphenol RSL74-0540-8 OpenCAPI cables (260 mm) were used to coherently connect the FPGA boards to the POWER9 CPUs. Finisar FTLC9558REPM 100GBASE-SR4 transceivers were used for the FPGA boards. For optimal performance, count of threads per core (simultaneous multithreading) was set to 2.

The configuration can be adapted based on experimental needs. For example, memory capacity should be adjusted for maximal length of burst operation, if uncompressed or poorly compressed images are to be collected at the full frame rate. The number of GPGPUs will affect frame rate for spot-finding operation, while the number of FPGA boards can be reduced to 1 if only small detectors are to be operated with the system.

### A2. Software requirements

Building Jungfraujoch software necessitates C++17 compliant compiler (*e.g.* GCC 11.2), *CMake* 3.15 or newer, HDF5 library 1.10 or newer, ZeroMQ library, Nvidia CUDA environment 11.5, as well as *gRPC*.

Building the FPGA design requires *Xilinx Vivado* 2022.1 and *Xilinx Vitis High-Level Synthesis* 2022.1. To synthesize all card features, license for the Alpha Data 9H3 reference design is necessary. *Cadence Xcelium* simulator 20.09 is needed to simulate the FPGA design.

### A3. FPGA design details

The current FPGA design uses roughly a quarter of the resources on the XCVU35P FPGA and half of its area (see Table 2 ▸ and Fig. 4 ▸), leaving space for future extensions, such as compression or on-the-fly analysis.

### A4. Source code

The source is publicly available from PSI GitLab at https://gitlab.psi.ch/jungfraujoch/jungfraujoch.

Software components are licensed under the GNU Public License version 3 and hardware components are licensed under the strongly reciprocal variant of CERN Open Hardware License. Synthesized FPGA bitstream for the Alpha Data 9H3 board is available from the authors upon request.

The code includes external libraries, specifically: *OC-Accel*, *OpenCAPI Simulation Engine*, *bitshuffle* (pre-)compression (Masui *et al.*, 2015 ▸), *Spdlog* for logging, *GEMMI* (Wojdyr, 2022 ▸), *tinycbor* (Intel), *C++ JSON parser* (Lohmann, 2022 ▸), *SLS Detector Package*, *XGANDALF* indexer (Gevorkov *et al.*, 2019 ▸), *Xilinx High-Level Synthesis* headers (arbitrary precision numbers and AXImm burst) and *Catch2* testing framework. Links to the source code repository for the libraries mentioned above can be found in the Jungfraujoch source code.

### A5. Design verification

A three-step procedure was developed to verify Jungfraujoch: two steps before deployment on the hardware platform and the last step after deployment. The first step was compiling high-level synthesis code with a standard C++ compiler and running a standard testing framework (*Catch2*), making it possible to verify all routines quickly. The second step was simulation with the help of the *OpenCAPI Simulation Engine* and *Cadence Xcelium*. Here, the whole FPGA design, in HDL and including the OpenCAPI interface, is simulated together with software (CPU/GPU) code running a short data collection. The speed of the hardware simulation is slow: simulating three frames of a single-module system takes roughly 2–3 h. These two tests were implemented with a continuous integration pipeline (GitLab), ensuring that bugs can be identified and fixed quickly. The last verification step, after deployment, is an application to test the card behavior and performance with FPGA hardware, but without a detector connected, using the FPGA internal packet generator.

### A6. JUNGFRAU detector

The JUNGFRAU detector is a pixel-array charge-integrating detector with a pixel pitch of 75 µm, which can operate up to a 2.2 kHz frame rate. JUNGFRAU is a modular detector, where each module (512 × 1024 pixels ≃ 500k pixels) is equipped with independent control (copper; 100 MB s^−1^) and readout (fiberoptic; 2 × 10 Gbit s^−1^) network connections. The JUNGFRAU pixel is always encoded with 16 bits. The JUNGFRAU 4M detector was used for testing Jungfraujoch. Further detector details can be found in our prior publications (Mozzanica *et al.*, 2018 ▸; Redford *et al.*, 2018*a* ▸,*b* ▸; Leonarski *et al.*, 2018 ▸, 2020 ▸).

## References

[bb1] Allan, D., Caswell, T., Campbell, S. & Rakitin, M. (2019). *Synchrotron Rad. News*, **32**(3), 19–22.

[bb2] Basu, S., Olieric, V., Leonarski, F., Matsugaki, N., Kawano, Y., Takashi, T., Huang, C.-Y., Yamada, Y., Vera, L., Olieric, N., Basquin, J., Wojdyla, J. A., Bunk, O., Diederichs, K., Yamamoto, M. & Wang, M. (2019). *IUCrJ*, **6**, 373–386. 10.1107/S2052252519002756 PMC6503925 31098019

[bb3] Bernstein, H. J., Förster, A., Bhowmick, A., Brewster, A. S., Brockhauser, S., Gelisio, L., Hall, D. R., Leonarski, F., Mariani, V., Santoni, G., Vonrhein, C. & Winter, G. (2020). *IUCrJ*, **7**, 784–792. 10.1107/S2052252520008672 PMC7467160 32939270

[bb4] Broennimann, Ch., Eikenberry, E. F., Henrich, B., Horisberger, R., Huelsen, G., Pohl, E., Schmitt, B., Schulze-Briese, C., Suzuki, M., Tomizaki, T., Toyokawa, H. & Wagner, A. (2006). *J. Synchrotron Rad.* **13**, 120–130. 10.1107/S0909049505038665 16495612

[bb5] Casanas, A., Warshamanage, R., Finke, A. D., Panepucci, E., Olieric, V., Nöll, A., Tampé, R., Brandstetter, S., Förster, A., Mueller, M., Schulze-Briese, C., Bunk, O. & Wang, M. (2016). *Acta Cryst.* D**72**, 1036–1048. 10.1107/S2059798316012304 PMC5013597 27599736

[bb711] Collet, Y. & Kucherawy, M. (2021) *Zstandard Compression and the application/zstd Media Type*, https://www.rfc-editor.org/rfc/rfc8878.

[bb6] Denes, P. & Schmitt, B. (2014). *J. Synchrotron Rad.* **21**, 1006–1010. 10.1107/S1600577514017135 PMC4181641 25177989

[bb7] Diederichs, K. & Wang, M. (2017). *Serial Synchrotron X-ray Crystallography (SSX)*, pp. 239–272. New York: Springer New York. 10.1007/978-1-4939-7000-1_10 28573576

[bb8] Dinapoli, R., Bergamaschi, A., Henrich, B., Horisberger, R., Johnson, I., Mozzanica, A., Schmid, E., Schmitt, B., Schreiber, A., Shi, X. & Theidel, G. (2011). *Nucl. Instrum. Methods Phys. Res. A*, **650**, 79–83.

[bb9] Durante, P., Neufeld, N., Schwemmer, R., Marconi, U., Balbi, G. & Lax, I. (2015). *IEEE Trans. Nucl. Sci.* **62**, 1752–1757.

[bb10] Förster, A., Brandstetter, S. & Schulze-Briese, C. (2019). *Philos. Trans. R. Soc. A*, **377**, 20180241. 10.1098/rsta.2018.0241 PMC6501887 31030653

[bb11] Gevorkov, Y., Yefanov, O., Barty, A., White, T. A., Mariani, V., Brehm, W., Tolstikova, A., Grigat, R.-R. & Chapman, H. N. (2019). *Acta Cryst.* A**75**, 694–704. 10.1107/S2053273319010593 PMC6718201 31475914

[bb12] Gibbons, E. P., Heron, M. T. & Rees, N. P. (2011). *Proceedings of the 13th International Conference on Accelerators and Large Experimental Physics Control Systems (ICALEPCS2011)*, 10–14 October 2011, Grenoble, France, pp. 529–532. TUAAUST01.

[bb13] Hauf, S., Heisen, B., Aplin, S., Beg, M., Bergemann, M., Bondar, V., Boukhelef, D., Danilevsky, C., Ehsan, W., Essenov, S., Fabbri, R., Flucke, G., Fulla Marsa, D., Göries, D., Giovanetti, G., Hickin, D., Jarosiewicz, T., Kamil, E., Khakhulin, D., Klimovskaia, A., Kluyver, T., Kirienko, Y., Kuhn, M., Maia, L., Mamchyk, D., Mariani, V., Mekinda, L., Michelat, T., Münnich, A., Padee, A., Parenti, A., Santos, H., Silenzi, A., Teichmann, M., Weger, K., Wiggins, J., Wrona, K., Xu, C., Youngman, C., Zhu, J., Fangohr, H. & Brockhauser, S. (2019). *J. Synchrotron Rad.* **26**, 1448–1461. 10.1107/S1600577519006696 31490132

[bb14] Hennessy, J. L. & Patterson, D. A. (2019). *Commun. ACM*, **62**, 48–60.

[bb15] Hoozemans, J., Peltenburg, J., Nonnemacher, F., Hadnagy, A., Al-Ars, Z. & Hofstee, H. P. (2021). *IEEE Circuits Syst. Mag.* **21**, 30–47.

[bb16] Kabsch, W. (2010). *Acta Cryst.* D**66**, 133–144. 10.1107/S0907444909047374 PMC2815666 20124693

[bb17] Kaminski, J. W., Vera, L., Stegmann, D., Vering, J., Eris, D., Smith, K. M. L., Huang, C.-Y., Meier, N., Steuber, J., Wang, M., Fritz, G., Wojdyla, J. A. & Sharpe, M. E. (2022). *Acta Cryst.* D**78**, 328–336. 10.1107/S2059798322000705 PMC8900825 35234147

[bb18] Ke, T.-W., Brewster, A. S., Yu, S. X., Ushizima, D., Yang, C. & Sauter, N. K. (2018). *J. Synchrotron Rad.* **25**, 655–670. 10.1107/S1600577518004873 PMC5929353 29714177

[bb19] Leonarski, F., Mozzanica, A., Brückner, M., Lopez-Cuenca, C., Redford, S., Sala, L., Babic, A., Billich, H., Bunk, O., Schmitt, B. & Wang, M. (2020). *Struct. Dyn.* **7**, 014305. 10.1063/1.5143480 PMC7044001 32128347

[bb20] Leonarski, F., Redford, S., Mozzanica, A., Lopez-Cuenca, C., Panepucci, E., Nass, K., Ozerov, D., Vera, L., Olieric, V., Buntschu, D., Schneider, R., Tinti, G., Froejdh, E., Diederichs, K., Bunk, O., Schmitt, B. & Wang, M. (2018). *Nat. Methods*, **15**, 799–804. 10.1038/s41592-018-0143-7 30275593

[bb40] Lohmann, N. (2022). *JSON for Modern C++*, https://github.com/nlohmann/json.

[bb21] Masui, K., Amiri, M., Connor, L., Deng, M., Fandino, M., Höfer, C., Halpern, M., Hanna, D., Hincks, A., Hinshaw, G., Parra, J., Newburgh, L., Shaw, J. & Vanderlinde, K. (2015). *Astron. Comput.* **12**, 181–190.

[bb22] Michel, V., Beteva, A., Coutinho, T. M., Dominguez, M. C., Guijarro, M., Guilloud, C., Homs, A., Meyer, J. M., Papillon, E., Perez, M. & Petitdemange, S. (2019). *Proceedings of the 12th International Workshop on Emerging Technologies and Scientific Facilities Controls (PCaPAC’18)*, 16–19 October 2018, Hsinchu, Taiwan, pp. 26–29. WEP02.

[bb23] Mozzanica, A., Andrä, M., Barten, R., Bergamaschi, A., Chiriotti, S., Brückner, M., Dinapoli, R., Fröjdh, E., Greiffenberg, D., Leonarski, F., Lopez-Cuenca, C., Mezza, D., Redford, S., Ruder, C., Schmitt, B., Shi, X., Thattil, D., Tinti, G., Vetter, S. & Zhang, J. (2018). *Synchrotron Radiat. News*, **31**(6), 16–20.

[bb24] Mueller, M., Wang, M. & Schulze-Briese, C. (2012). *Acta Cryst.* D**68**, 42–56. 10.1107/S0907444911049833 PMC3245722 22194332

[bb25] Nakamura, H., Takayama, H., Yamaguchi, Y. & Boku, T. (2017). *Proceedings of the 2017 International Conference on ReConFigurable Computing and FPGAs (ReConFig)*, 4–6 December 2017, Cancun, Mexico, pp. 1–6.

[bb26] Oscarsson, M., Beteva, A., Flot, D., Gordon, E., Guijarro, M., Leonard, G., McSweeney, S., Monaco, S., Mueller-Dieckmann, C., Nanao, M., Nurizzo, D., Popov, A., von Stetten, D., Svensson, O., Rey-Bakaikoa, V., Chado, I., Chavas, L., Gadea, L., Gourhant, P., Isabet, T., Legrand, P., Savko, M., Sirigu, S., Shepard, W., Thompson, A., Mueller, U., Nan, J., Eguiraun, M., Bolmsten, F., Nardella, A., Milàn-Otero, A., Thunnissen, M., Hellmig, M., Kastner, A., Schmuckermaier, L., Gerlach, M., Feiler, C., Weiss, M. S., Bowler, M. W., Gobbo, A., Papp, G., Sinoir, J., McCarthy, A., Karpics, I., Nikolova, M., Bourenkov, G., Schneider, T., Andreu, J., Cuní, G., Juanhuix, J., Boer, R., Fogh, R., Keller, P., Flensburg, C., Paciorek, W., Vonrhein, C., Bricogne, G. & de Sanctis, D. (2019). *J. Synchrotron Rad.* **26**, 393–405. 10.1107/S1600577519001267 PMC6412183 30855248

[bb27] Petitdemange, S., Claustre, L., Homs, A., Regojo, R. H., Papillon, E., Langlois, F., Mant, G. R. & Noureddine, A. (2014). *Proceedings of the 16th International Conference on Accelerator and Large Experimental Physics Control Systems (ICALEPCS2017)*, 8–13 October 2017, Barcelona, Spain, pp. 886–890. TUPHA194.

[bb28] Redford, S., Andrä, M., Barten, R., Bergamaschi, A., Brückner, M., Chiriotti, S., Dinapoli, R., Fröjdh, E., Greiffenberg, D., Leonarski, F., Lopez-Cuenca, C., Mezza, D., Mozzanica, A., Ruder, C., Schmitt, B., Shi, X., Thattil, D., Tinti, G., Vetter, S. & Zhang, J. (2018*b*). *J. Instrum.* **13**, C11006.

[bb29] Redford, S., Andrä, M., Barten, R., Bergamaschi, A., Brückner, M., Dinapoli, R., Fröjdh, E., Greiffenberg, D., Lopez-Cuenca, C., Mezza, D., Mozzanica, A., Ramilli, M., Ruat, M., Ruder, C., Schmitt, B., Shi, X., Thattil, D., Tinti, G., Vetter, S. & Zhang, J. (2018*a*). *J. Instrum.* **13**, C01027.

[bb30] Roy, R., Sato, K., Bhattachrya, S., Fang, X., Joti, Y., Hatsui, T., Hiraki, T. N., Guo, J. & Yu, W. (2021). *21st IEEE/ACM International Symposium on Cluster, Cloud and Internet Computing (CCGrid 2021)*, 10–13 May 2021, Melbourne, Australia, pp. 41–50.

[bb31] Ruiz, M., Sidler, D., Sutter, G., Alonso, G. & López-Buedo, S. (2019). *Proceedings of the 29th International Conference on Field-Programmable Logic and Applications (FPL 2019)*, 9–13 September 2019, Barcelona, Spain, pp. 286–292.

[bb32] Stuecheli, J., Starke, W. J., Irish, J. D., Arimilli, L. B., Dreps, D., Blaner, B., Wollbrink, C. & Allison, B. (2018). *IBM J. Res. Dev.* **62**, 8:1–8:8.

[bb33] Sutter, G., Ruiz, M., López-Buedo, S. & Alonso, G. (2018). *Proceedings of the 2018 International Conference on ReConFigurable Computing and FPGAs (ReConFig)*, 3–5 December 2018, Cancun, Mexico.

[bb34] Thomas, S. E., Collins, P., James, R. H., Mendes, V., Charoensutthivarakul, S., Radoux, C., Abell, C., Coyne, A. G., Floto, R. A., von Delft, F. & Blundell, T. L. (2019). *Philos. Trans. R. Soc. A*, **377**, 20180422. 10.1098/rsta.2018.0422 PMC6501894 31030650

[bb35] Wang, Z., Huang, H., Zhang, J. & Alonso, G. (2020). *Proceedings of the 2020 IEEE 28th Annual International Symposium on Field-Programmable Custom Computing Machines (FCCM)*, 3–6 May 2020, Fayetteville, AR, USA, pp. 111–119.

[bb712] Wojdyr M. (2022) *J. Open Source Softw.* **7**, 4200.

[bb36] Wojdyla, J. A., Kaminski, J. W., Panepucci, E., Ebner, S., Wang, X., Gabadinho, J. & Wang, M. (2018). *J. Synchrotron Rad.* **25**, 293–303. 10.1107/S1600577517014503 PMC5741135 29271779

